# Supplementary material for: γδ T cells control humoral immune response by inducing T follicular helper cell differentiation
Source: Nat Commun. 2018 Aug 8;9:3151. doi: 10.1038/s41467-018-05487-9 (PMC6082880; doi:10.1038/s41467-018-05487-9)
Supplement: Supplementary file 1 — Supplementary Information [file 41467_2018_5487_MOESM1_ESM.pdf]

γδ T cells control humoral immune response by inducing T follicular helper cell differentiation

Rezende et al.

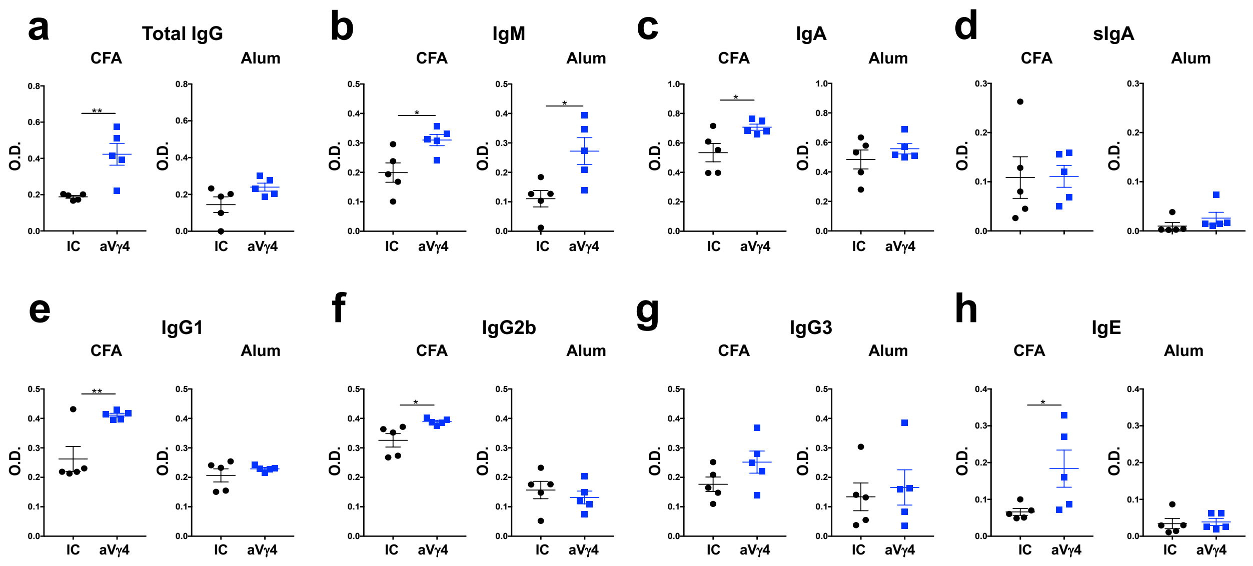

Supplementary Figure 1. Differential involvement of γδ T cell subsets on OVA-specific antibody production. **(a-h)** Serum and fecal (secretory IgA) OVA-specific total IgG **(a)**, IgM **(b)**, IgA **(c)**, secretory IgA (sIgA; **d)**, IgG1 **(e)**, IgG2b **(f)**, IgG3 **(g)** and IgE **(h)** from WT mice treated with either anti-Vγ4 depleting monoclonal antibody (aVγ4) or isotype control (IC) three days before s.c. immunization with either OVA+CFA or OVA+Alum and three days before an OVA booster dose. Mice received a boost of OVA 14 days after immunization (n=5 mice/group). Data are shown as mean + SEM. One-way ANOVA was used. NS= non-significant, * p<0.05, ** p<0.01.

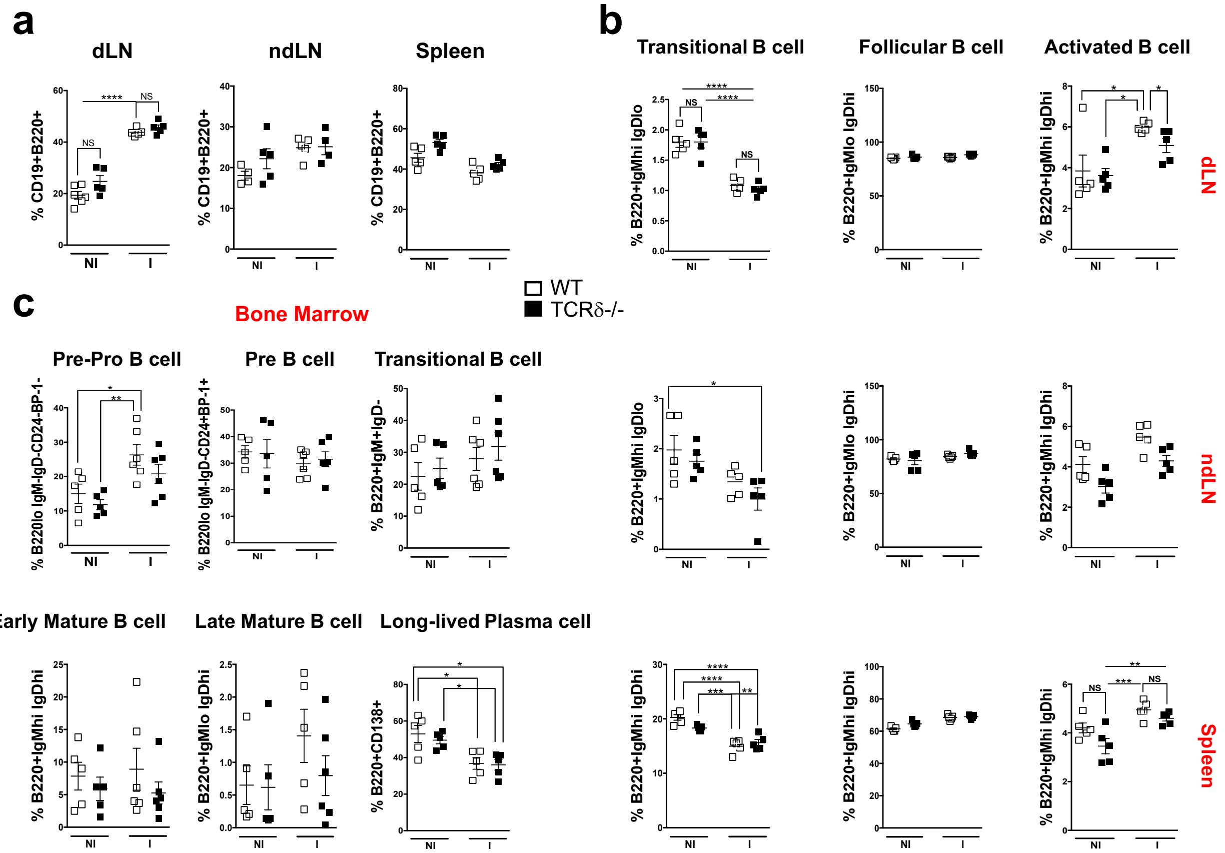

Supplementary Figure 2. B cell compartment is not impaired in TCRδ-/- mice. **(a)** Frequency of total B cells (CD19+B220+) in dLN, ndLN and spleen of non-immunized (NI) WT and TCRδ-/- mice and 7 days after CFA immunization (I) (n=4-6 mice/group). **(b)** Frequency of Transitional (B220+IgM^{hi}IgD^{lo}), Follicular (B220+IgM^{lo}IgD^{hi}) or Activated (B220+IgM^{hi}IgD^{hi}) B cells in dLN, ndLN and spleen of non-immunized (NI) WT and TCRδ-/- mice and 7 days after CFA immunization (I) (n=5 mice/group). **(c)** Frequency of Pre-Pro (B220^{lo}IgM-IgD-CD24-BP-1-), Pre (B220^{lo}IgM-IgD-CD24+BP-1+), Transitional (B220+IgM+IgD-), Early Mature (B220+IgM^{hi}IgD^{hi}), Late Mature (B220+IgM^{lo}IgD^{hi}) B cells and long-lived plasma cells (B220+CD138+) from bone marrow of non-immunized (NI) WT and TCRδ-/- mice and 7 days after CFA immunization (I) (n=5 mice/group). These data are representative of 2 independent experiments. Data are shown as mean ± SEM. One-way ANOVA was used. NS= non-significant, * p<0.05, ** p<0.01, *** p<0.001, **** p<0.0001.

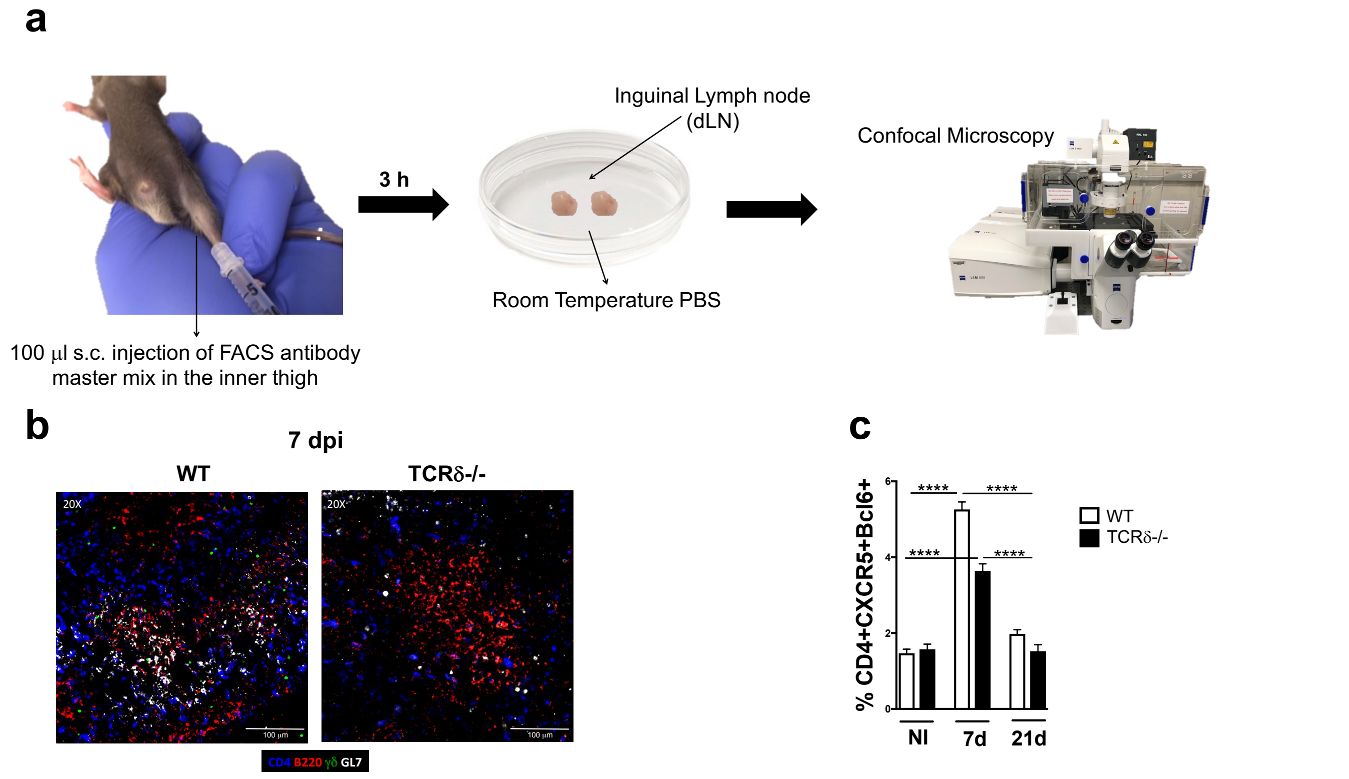

Supplementary Figure 3. Germinal center formation is impaired in TCRδ-/- mice. **(a)** Protocol for whole lymph node confocal microscopy. Mice were injected s.c. in the thigh with 20 μl of a master mix of FACS antibodies and 3h later they were sacrificed and inguinal lymph nodes (dLN) collected for confocal microscopy. **(b)** Representative confocal microscopy images of whole lymph nodes (dLN) of non-immunized (NI) TCRγδ-GFP and TCRδ-/- mice, and 7 days post CFA immunization (dpi). Scale bar=100 μm. CD4-blue; B220-red; TCRγδ-green; GL7-gray (n=5 mice/group). **(c)** Frequency of CD4+ T cells expressing CXCR5 and Bcl6 in the dLNs of non-immunized (NI) WT and TCRδ-/- mice and 7 and 21 days post CFA immunization (n=6 mice/group). These data are representative of 2 independent experiments. Data are shown as mean ± SEM. One-way ANOVA was used. **** $p<0.0001$.

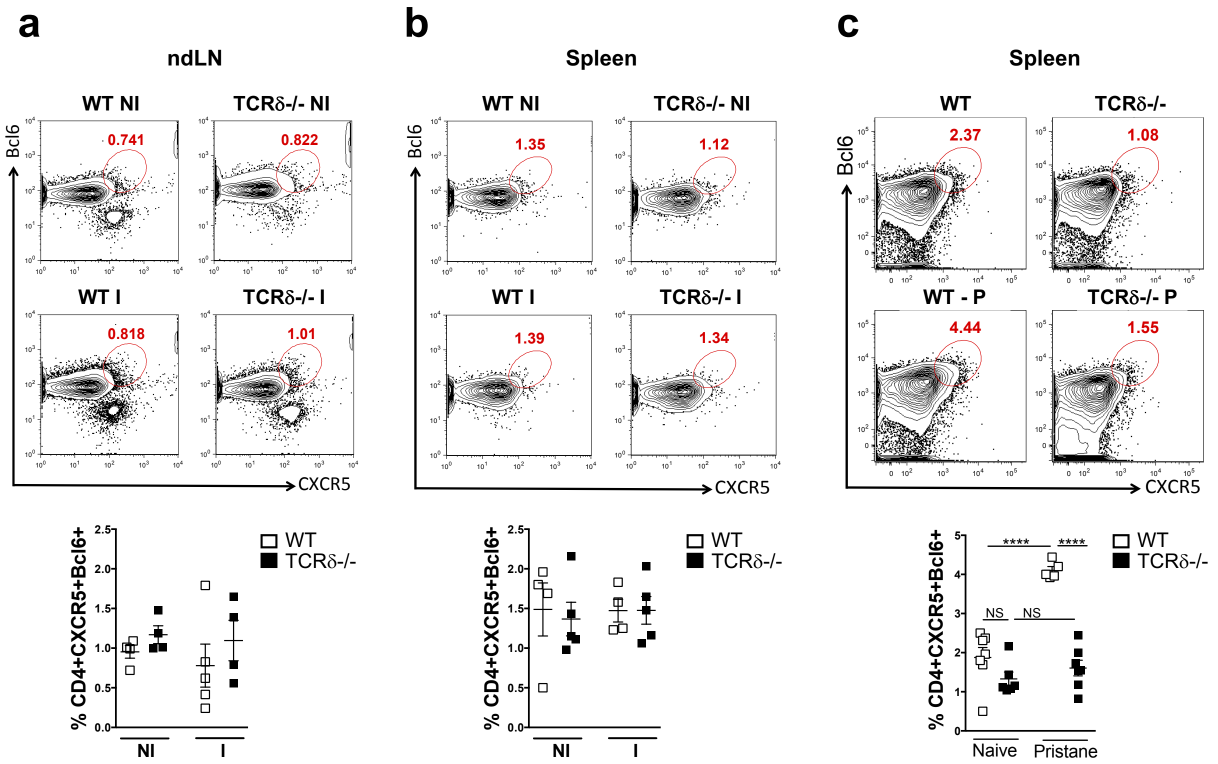

Supplementary Figure 4. Tfh cell compartment in TCRδ-/- mice. **(a, b)** Frequency of CD4+ T cells expressing CXCR5 and Bcl6 in ndLN (**a**) and spleen **(b)** of non-immunized (NI) WT and TCRδ-/- mice and 7 days after CFA immunization (I) (n=4-5 mice/group). **(c)** Frequency of CD4+ T cells expressing CXCR5 and Bcl6 in the spleen of naïve WT and TCRδ-/- mice and 5 months after pristane injection (WT-P, TCRδ-/- P) (n=5-8 mice/group). These data are representative of at least 2 independent experiments. Data are shown as mean ± SEM. One-way ANOVA was used. NS= non-significant, *** $p<0.001$, **** $p<0.0001$.

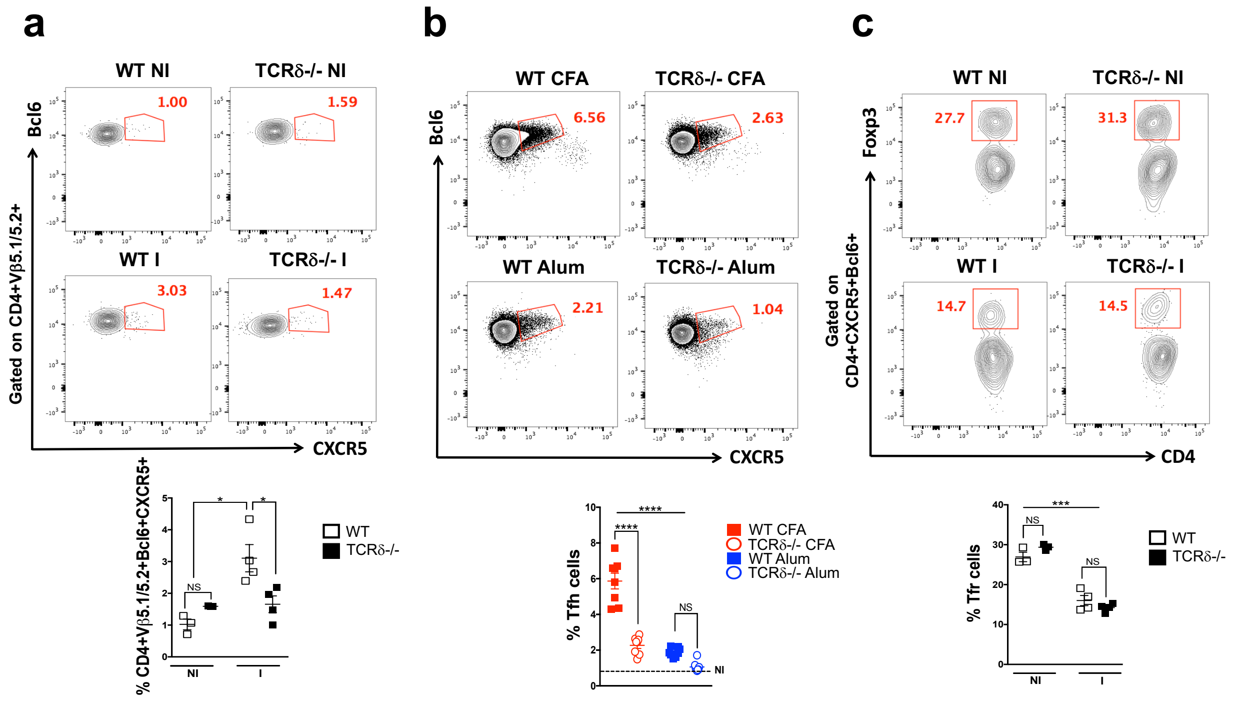

Supplementary Figure 5. CFA-immunized TCRδ-/- mice have reduced OVA-specific Tfh cells, but normal Tfr cell compartment. **(a)** Frequency of TCRVβ5.1/5.2 CD4+ T cells expressing CXCR5 and Bcl6 in dLN of non-immunized (NI) WT and TCRδ-/- mice and 7 days after CFA immunization (I) (n=3-4 mice/group). **(b)** Frequency of CD4+ T cells expressing CXCR5 and Bcl6 in dLN from WT and TCRδ-/- mice 7 days after either CFA or Alum immunization (n=8 mice/group). **(c)** Frequency of Foxp3 expression in CD4+CXCR5+Bcl6+ (T follicular regulatory, Tfr) cells in the dLN of non-immunized (NI) WT and TCRδ-/- mice and 7 days after CFA immunization (I) (n=3-4 mice/group). These data are representative 2 independent experiments. Data are shown as mean ± SEM. One-way ANOVA was used. NS= non-significant, * $p<0.05$, *** $p<0.001$, **** $p<0.0001$.

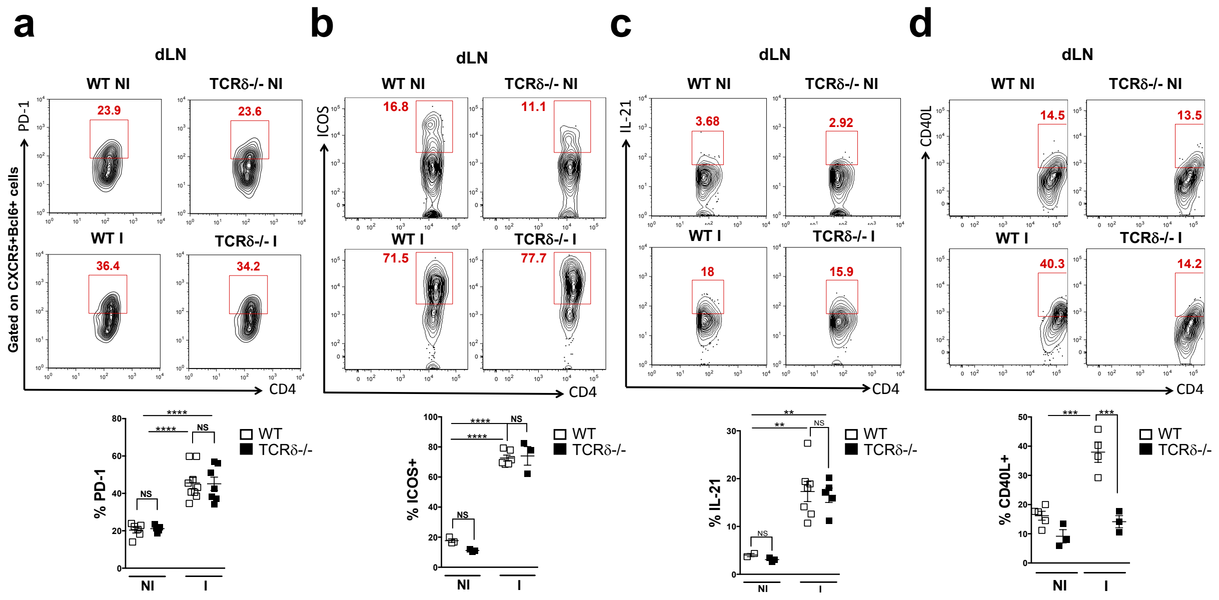

Supplementary Figure 6. Characterization of Tfh cell in WT vs. TCRδ-/- mice. **(a-d)** Frequency of Tfh cells expressing PD-1 **(a)**, ICOS **(b)**, IL21 **(c)** and CD40L **(d)** in dLN of non-immunized (NI) WT and TCRδ-/- mice and 7 days after CFA immunization (I) (n=3-9 mice/group). These data are representative of 3 independent experiments. Data are shown as mean + SEM. One-way ANOVA was used. NS= non-significant, ** p<0.01, *** p<0.001, **** p<0.0001.

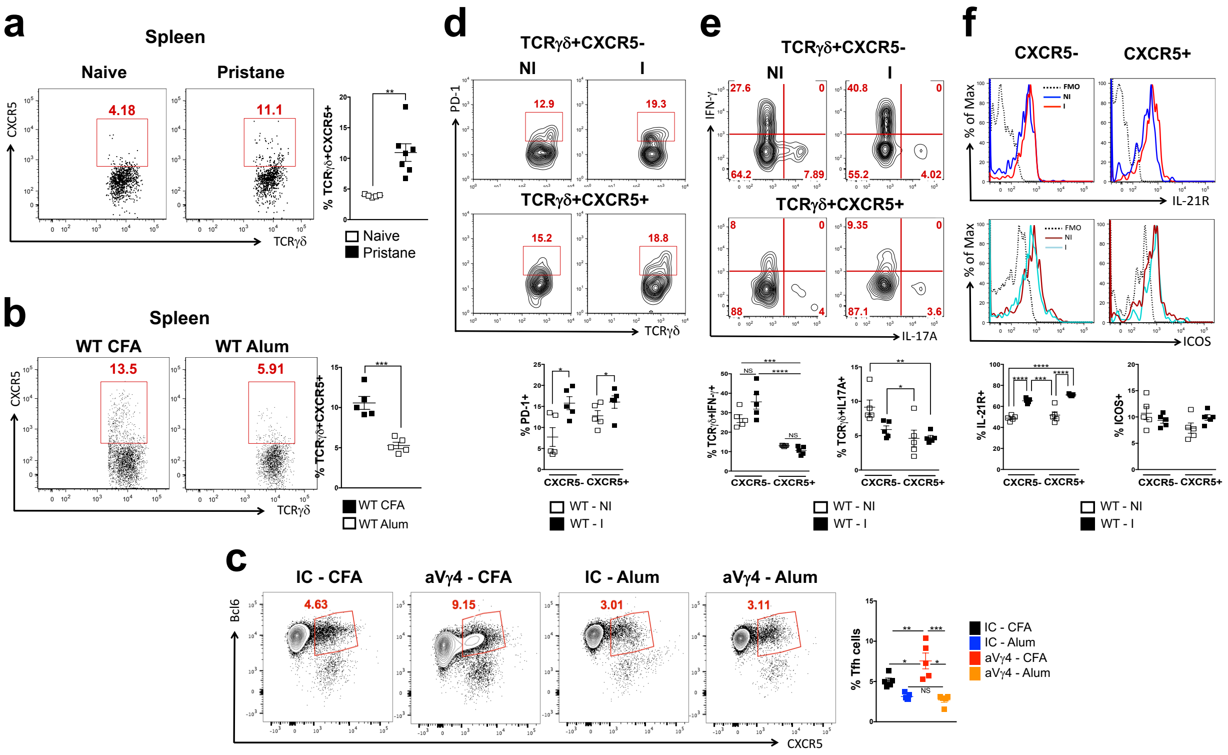

Supplementary Figure 7. CXCR5-expressing γδ T cells share some features with Tfh cells. **(a)** Frequency of γδ T cells expressing CXCR5 in the spleen of naïve WT mice and 5 months after pristane injection (Pristane) (n=6 mice/group). **(b)** Frequency of γδ T cells expressing CXCR5 in the spleen of WT and TCRδ-/- mice 21 days after s.c. immunization with either CFA/OVA or Alum/OVA. Mice received a boost of OVA 14 days after immunization (n=5 mice/group). **(c)** Frequency of CD4+ T cells expressing CXCR5 and Bcl6 in the spleen from mice treated with either anti-Vγ4 mAb (aVγ4) or isotype control (IC) 21 days post CFA/OVA or Alum/OVA immunization (n=5 mice/group). **(d)** Frequency of PD-1-expressing TCRγδ+CXCR5- and TCRγδ+CXCR5+ cells from dLN of non-immunized (NI) WT mice and 7 days after CFA immunization (I) (n=5 mice/group). **(e)** Frequency of IFN-γ and IL-17A-expressing TCRγδ+CXCR5- and TCRγδ+CXCR5+ cells from dLN of non-immunized (NI) WT mice and 7 days after CFA immunization (I) (n=5 mice/group). **(f)** Histogram and frequency of IL-21 receptor (IL-21R) and ICOS-expressing TCRγδ+CXCR5- and TCRγδ+CXCR5+ cells from dLN of non-immunized (NI) WT mice and 7 days after CFA immunization (I) (n=5 mice/group). These data are representative of 2 independent experiments. Data are shown as mean ± SEM. Student's *t*-test **(a, b)** and One-way ANOVA **(c-e)** were used. * $p<0.05$, ** $p<0.01$, *** $p<0.001$, **** $p<0.0001$.

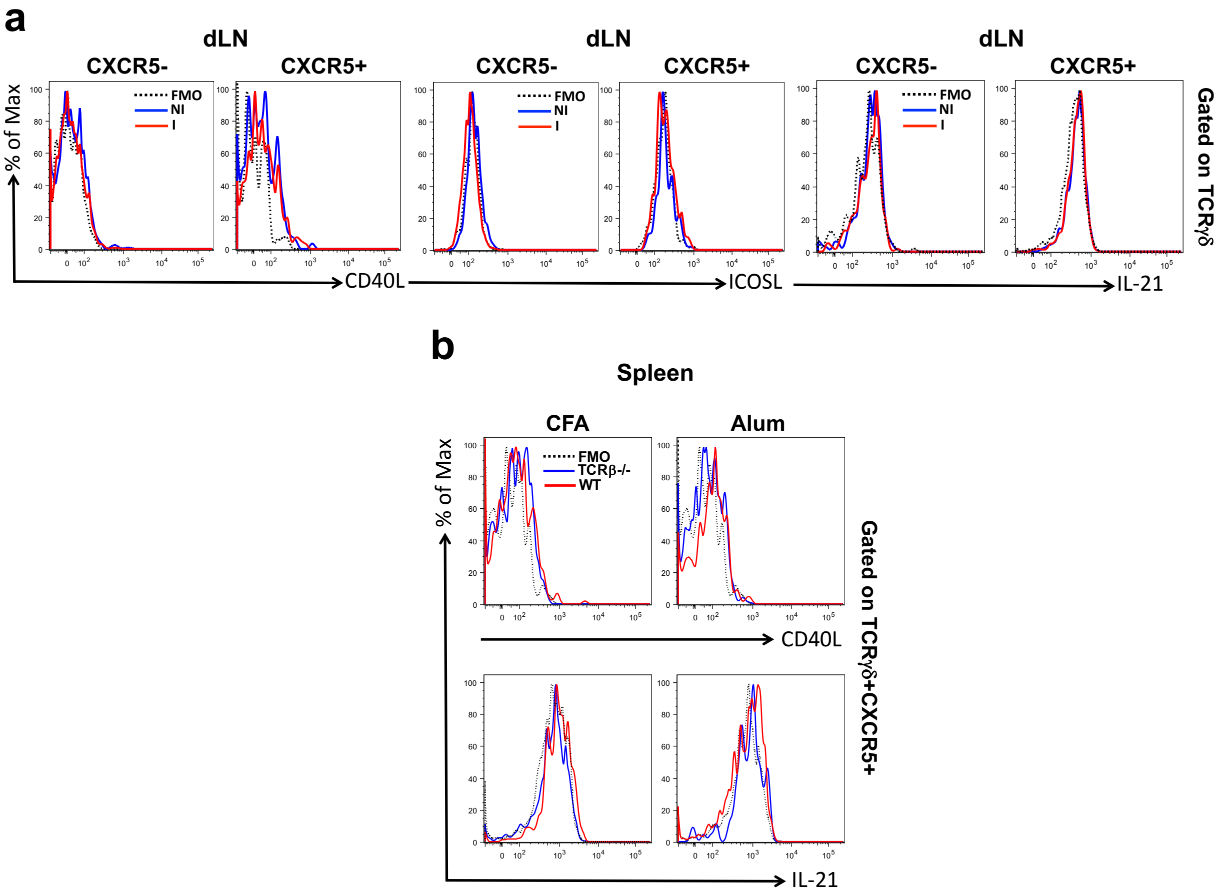

Supplementary Figure 8. CXCR5-expressing γδ T cells do not directly help B cells. **(a)** Representative histograms of TCRγδ+CXCR5- and TCRγδ+CXCR5+ cells expressing CD40L, ICOSL and IL-21 in dLN of non-immunized (NI) WT mice and 7 days after CFA immunization (I) (n=3-5 mice/group). **(b)** Representative histograms of TCRγδ+CXCR5+ cells from spleen of WT, TCRβ-/- and TCRδ-/- mice expressing CD40L and IL-21, 21 days after s.c. immunization with either CFA/OVA or Alum/OVA. Mice received a boost of OVA 14 days after immunization (n=5 mice/group).

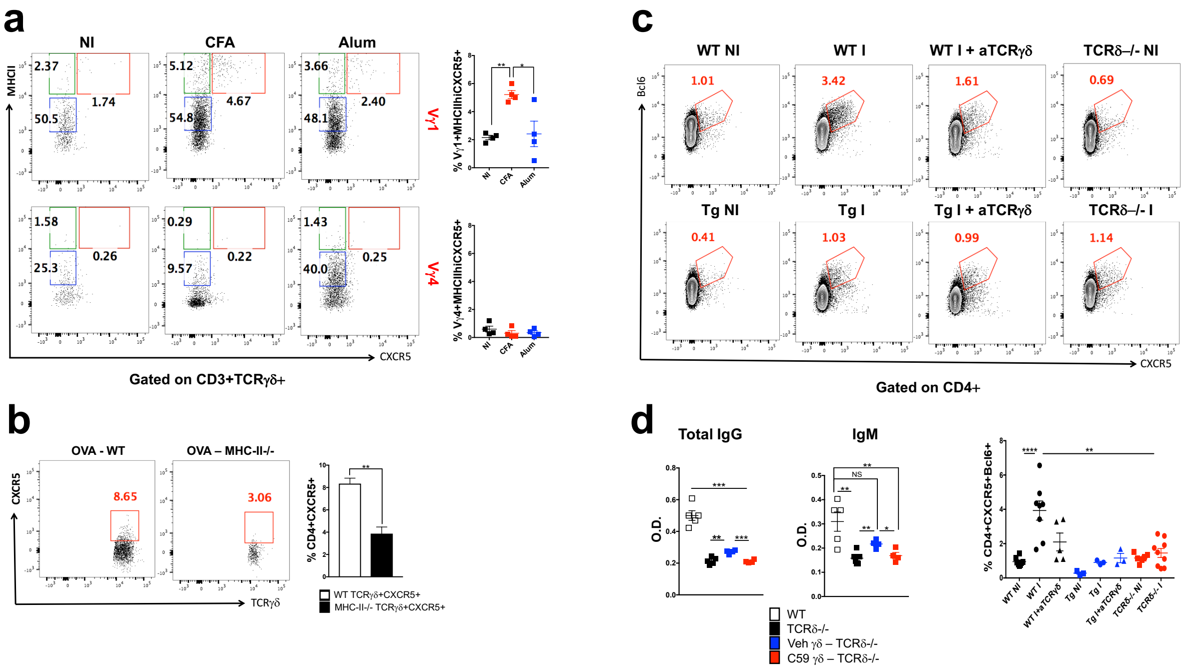

Supplementary Figure 9. β-catenin-dependent control of antibody production by γδ T cells, antigen presentation properties of TCRγδ+CXCR5+ cells and TCF-1 involvement in Tfh cell differentiation. **(a)** Frequency of Vγ1 and Vγ4 γδ T cells expressing CXCR5 and MHC-II from dLN of non-immunized (NI) WT mice and 3 days after either CFA or Alum immunization (n=4 mice/group). **(b)** CXCR5 expression on CellTrace Violet-stained naïve CD4 T cells from OT-II-Foxp3-GFP mice co-cultured (3 days at 37°C) with or without $OVA_{323-339}$-loaded TCRγδ+CXCR5+ from dLN of WT or MHC-II-/- mice 4 days after CFA immunization (n=pooled cells from 10 mice/experiment). **(c)** Frequency of CD4+ T cells expressing CXCR5 and Bcl6 in dLNs of non-immunized (NI) WT, Tcf7L Tg and TCRδ-/- mice and 7 days after CFA immunization (I). Mice were treated with either anti-TCRγδ (aTCRγδ; 250 μg, i.p.) or isotype control 7 days before CFA immunization (n=3-8 mice/group). **(d)** Serum OVA-specific total IgG and IgM from WT, TCRδ-/- and TCRδ-/- mice transferred with γδ T cells from WT mice treated with either 10 mg/ml of Wnt-C59 (C59) or vehicle (Veh). WT mice were sacrificed 24h after Wnt-C59 or vehicle treatment and a total of $5x10^5$ γδ T cells were intravenously injected into TCRδ-/- mice, which were then immunized with CFA, boosted with OVA 14 days later and sacrificed 7 days thereafter (n=4 mice/group). These data are representative of at least 2 independent experiments. Data are shown as mean ± SEM. Two-way ANOVA **(a)**, One-way ANOVA (**c, d**) or Student's *t*-test **(b)** were used. * p<0.05, ** p<0.01, *** p<0.001, **** p<0.0001.

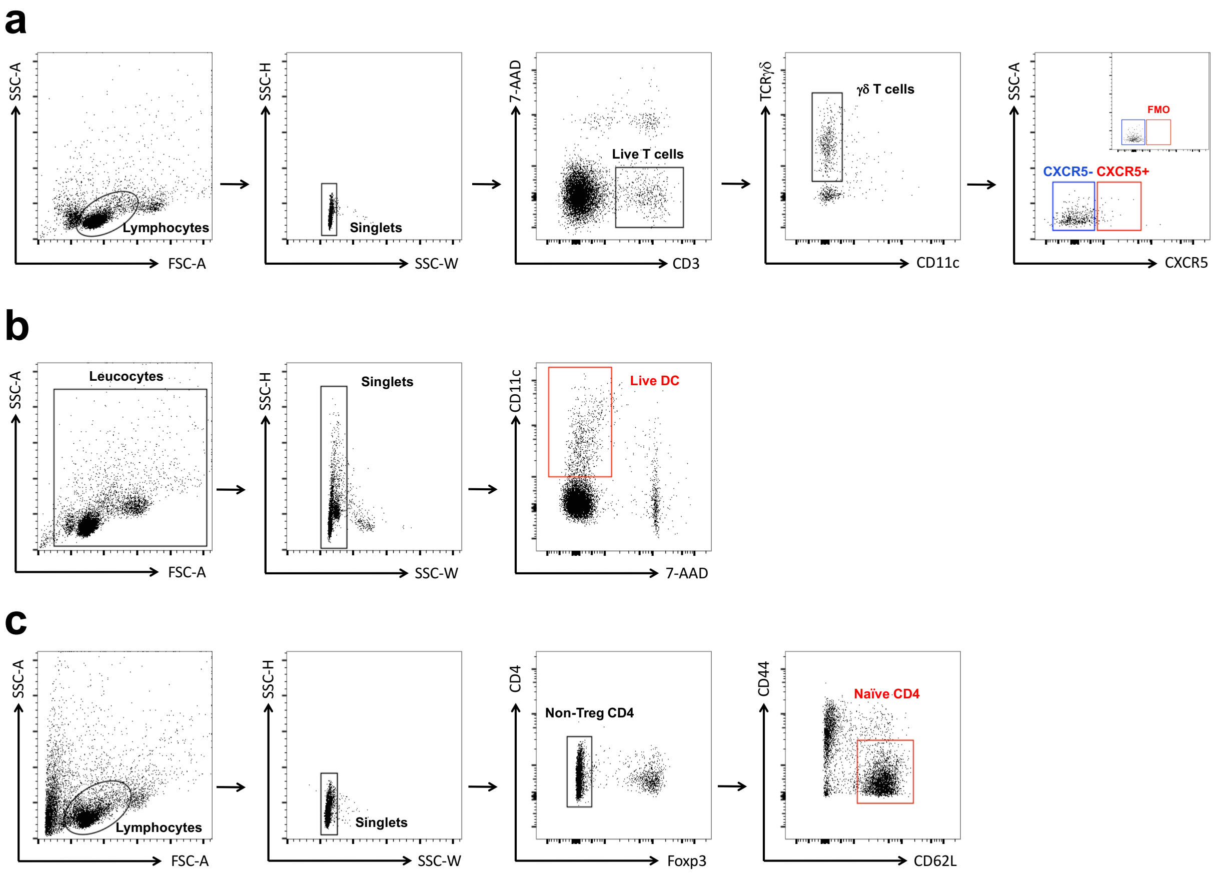

Supplementary Figure 10. Gating strategies for cell sorting. **(a)** Representative dot plots used for CXCR5- and CXCR5+ γδ T cell sorting. Inset dot plot shows the fluorescence minus one (FMO) control for CXCR5 staining. **(b)** Representative dot plots used for CD11c+ dendritic cell (DC) sorting. **(c)** Representative dot plots used for naïve CD4 T cell sorting.
