# Supplementary material for: γδ T cells control humoral immune response by inducing T follicular helper cell differentiation
Source: Nat Commun. 2018 Aug 8;9:3151. doi: 10.1038/s41467-018-05487-9 (PMC6082880; doi:10.1038/s41467-018-05487-9)
Supplement: Supplementary file 3 — Description of Additional Supplementary Files [file 41467_2018_5487_MOESM3_ESM.pdf]

Description of Additional Supplementary Files

File Name: Supplementary Data 1
Description: IgM and IgG autoantibody arrays comparing WT vs. TCRδ-/- mice before pristane injection (BPI; naïve) and 3 months after pristane injection (3MPI).

File Name: Supplementary Data 2
Description: Differential gene expression between TCRγδ+CXCR5+ vs. TCRγδ+CXCR5- cells - $p<0.05$.
